# Superior Cross-Species Reference Genes: A Blueberry Case Study

**DOI:** 10.1371/journal.pone.0073354

**Published:** 2013-09-18

**Authors:** Jose V. Die, Lisa J. Rowland

**Affiliations:** U.S. Department of Agriculture, Agricultural Research Service, Beltsville, Maryland, United States of America; United States Department of Agriculture, United States of America

## Abstract

The advent of affordable Next Generation Sequencing technologies has had major impact on studies of many crop species, where access to genomic technologies and genome-scale data sets has been extremely limited until now. The recent development of genomic resources in blueberry will enable the application of high throughput gene expression approaches that should relatively quickly increase our understanding of blueberry physiology. These studies, however, require a highly accurate and robust workflow and make necessary the identification of reference genes with high expression stability for correct target gene normalization. To create a set of superior reference genes for blueberry expression analyses, we mined a publicly available transcriptome data set from blueberry for orthologs to a set of *Arabidopsis* genes that showed the most stable expression in a developmental series. In total, the expression stability of 13 putative reference genes was evaluated by qPCR and a set of new references with high stability values across a developmental series in fruits and floral buds of blueberry were identified. We also demonstrated the need to use at least two, preferably three, reference genes to avoid inconsistencies in results, even when superior reference genes are used. The new references identified here provide a valuable resource for accurate normalization of gene expression in *Vaccinium* spp. and may be useful for other members of the *Ericaceae* family as well.

## Introduction

It has now been 20 years since the initial papers were published describing real-time quantitative PCR (qPCR, [1]). Since that time, it has become one of the most popular techniques in modern molecular biology. Once the technology was adopted by the research community, its use (measured as number of citations) increased dramatically with a growth curve resembling the sigmoidal amplification plots that are obtained during the qPCR analysis itself [2].

Today, qPCR is commonly considered a simple and mature technique; it is often referred to as the gold standard for mRNA quantification. However, it would actually be more accurate to define the technique in nearly the opposite terms. It is not really a mature technique. It is a versatile, evolving technology where new equipment, new chemistries, and even new algorithms are still being developed. The workflow is not simple or straightforward but requires a combination of various steps that have a direct impact on the accuracy of the results and the reliability of the conclusions [3], [4]. It is not a gold standard either. Inexperienced users, without the proper training, can produce results with nice-shaped sigmoidal amplification curves, even when important procedural controls are lacking, resulting in data of uncertain quality [3].

Conflicting results and difficulty replicating qPCR experiments may be the underlying reasons why semi-quantitative methods are still sometimes chosen in analyses today to estimate gene expression levels [5]. It is not that qPCR is intrinsically inaccurate, but rather it is the lack of a systematic procedure and performance that can lead to erroneous results and conclusions. Thus, one decade ago, increased awareness of problems associated with producing high-quality and reliable data from qRT-PCR came to the forefront [6]. Certainly, amongst the most important issues noted as affecting reproducibility was data normalization and proper selection of reference genes [7], [8], [9], [10]. Since then, the plant scientific community has gradually recognized the potential highly misleading effects of using inappropriate references for data normalization and how these effects compromise the interpretation of results [11], [12], [13], [14].

To overcome these issues, the use of one or more stable reference genes in qPCR has progressed in tandem with the evolution of mathematical equations for quantification, moving from the 1-reference model [15], [16] to modern advanced relative quantification models with multiple reference genes [17]. In April 2005, the “3rd London qPCR Symposium” determined that normalization against three or more validated reference genes was the most appropriate and universally applicable method [3]. This recommendation was further extended to include the set of qPCR best-practice guidelines that establishes the minimum experimental data necessary for evaluating qPCR quidelines, MIQE [18]. Furthermore, a set of MIQE key parameters recently highlighted the necessity of experimentally validating the utility of reference genes used during normalization, and the use of more than one validated reference gene is now described as an essential component of a reliable qPCR assay [19].

In the plant research community, more and more large-scale, high throughput gene expression studies are being conducted today in actual crop species, because of the advent of affordable Next Generation Sequencing (NGS) technologies. Interestingly, qPCR is still the method of choice for validation of gene expression changes detected in studies like RNA-Seq and microarrays. For many of these crops and experimental conditions, however, good reference genes are not known. For example, blueberry is an important fruit crop because of its high nutritional value, and it is one of the major berry crops grown in the United States [20]. There is interest in studying gene expression during fruit development that relates to general fruit quality and to nutritional value. There is also interest in using blueberry to study changes in gene expression during flower bud development associated with cold acclimation and chill unit accumulation [21]. Moreover, blueberry could serve as a model organism for the heath family *Ericaceae* in general which includes the economically important, closely related cranberry and lingonberry fruit crops, as well as the ornamentals, rhododendron, azalea, and mountain laurel. For many of these species, access to genomic technologies and genome-scale data sets is limited or completely lacking. Just recently, the first blueberry transcriptome, generated by NGS, became publicly available [22]; http://bioinformatics.towson.edu/BBGD454/) making possible computational analyses of thousands of sequences within the blueberry and related species. The transcriptome database is a valuable resource for identifying genes that may be differentially expressed and play important roles in plant and organ development, such as fruit development, and in abiotic and biotic stress responses [22]. The use of good reference genes as internal controls in these gene expression studies is absolutely essential, however. In addition to identifying differentially expressed genes, the blueberry database could also be mined for genes that are stably expressed, thus good candidate reference genes for data normalization, and in this sense, could be used in the same way that microarray data sets were exploited in the past [23], [24], [25]

A global transcriptomal comparison of different developmental stages of *Arabidopsis* has found new stably expressed reference genes with superior stability to other commonly used control genes for transcript normalization [26]. To create a set of genes for blueberry expression analyses, we have made use of the extensive amount of transcriptome data that is now publicly available and found orthologs of the highest stably expressed *Arabidopsis* genes. Using this strategy, we identified two sets of reference genes for flower buds and fruit, which provide a good starting point for accurate normalization of blueberry experiments. Additionally, we carried out a comprehensive evaluation of the weakness of a single reference gene approach for normalization. Finally, using a gene with important roles in fruit ripening, we found that the correct quantification of expression depends on the adoption of superior reference genes; otherwise, artifacts caused by the normalization process itself may result in erroneous conclusions. We anticipate these internal standards will provide a good foundation for evaluation of stably expressed genes in other woody perennials and related members of the *Ericaceae* family.

## Materials and Methods

### Plant material

Flower buds, fruits, and leaves were collected at multiple times during development from multiple plants of the highbush blueberry cultivar Bluecrop (*V. corymbosum*) and the southern rabbiteye cultivar Tifblue (*V. virgatum*), both grown at the USDA/ARS, Beltsville Agricultural Research Center, Beltsville, MD. The sample pools from each cultivar and each time point were made from a minimum of five plants. Flower buds were collected from field plants during the fall and winter of 2010–2011 with increasing exposure to chilling temperatures, measured as chill units (hours between 0–7°C). Buds were harvested at 0 (October, 10^th^), 370 (December, 3^rd^), 785 (January, 20^th^) and 1212 (March, 2^nd^) chill units. Fruit samples were collected from field plants during the 2008 growing season at four stages of ripening: green (June, 6^th^), white (June, 27^th^), pink (July, 7^th^) and blue or ripe (July, 7^th^). Leaves were collected every 3 weeks during the 2012 summer (Jun 22^nd^, July 16^th^, August 5^th^, August 30^th^ and Sept 21^st^). All tissues were frozen in liquid nitrogen immediately after harvest and stored at −80°C.

### RNA extraction and quality controls

For total RNA isolation, the leaf, fruit and bud samples were ground in liquid nitrogen and incubated at 65°C in a pre-warmed CTAB extraction buffer. Two or three chloroform∶IAA (24∶1) extractions were performed followed by overnight precipitation with LiCl [27]. RNA pellets were resuspended in DEPC-treated water, precipitated again with ethanol and NaOAc, washed, and finally resuspended in 1 ml DEPC-treated water. RNA concentration was determined by measuring the optical density at 260 nm using a NanoDrop ND-1000 spectrophotometer (Nanodrop Technologies, USA). RNA quality was assessed by combining information from several control steps. First, purity was inferred from the absorption ratios using the NanoDrop. Only the RNA samples with *A*_260_/*A*_280_ ratio between 1.9 and 2.1 (floral buds) and 1.75 and 2.1 (fruits) and *A*_260_/*A*_230_ greater than 2.0 were used in the analysis. Then, RNA samples were visualized on 1% agarose gels stained with ethidium bromide. Finally, we amplified segments of the 5′ and 3′ regions of a ubiquitin carboxyl-terminal hydrolase gene across the cDNA samples [28] by qPCR, as described below.

### First strand cDNA synthesis and quality controls

RNA extracts were treated with TURBO™ DNase I (Life Technologies, USA), prior to cDNA synthesis. The extracted RNA was split into 2 reverse transcription reactions. Two micrograms (floral buds and leaves) and ∼1 microgram (fruits) of DNase I-treated total RNA were used for the synthesis of cDNA. Complementary DNAs was synthesized by priming with oligo-dT_12–18_ (Life Technologies, USA), using SuperScriptIII reverse transcriptase (Life Technologies, USA) following the instructions of the provider. The cDNAs were diluted to a final volume of 100 µl. Each cDNA sample was then tested for presence of genomic DNA contamination (gDNA) by performing conventional PCR with a primer pair designed from two different exons of an alcohol dehydrogenase-like blueberry sequence (CF811586). The primer pair was designed to amplify a product of 1,140 bp using genomic DNA as template or 528 bp using cDNA as template (primers: NA799F, 5′-CCGCTGGTGATTGAAGAAGT-3′; NA799R, 5′-TTTCGCAACATTTAGCATGG-3′). In tests for gDNA contamination, the 1,140 bp band was not amplified from any of the samples. For qualitative assessment of the reverse transcriptase reaction and the RNA integrity (the efficiency of cDNA synthesis is dependent on the intactness of mRNA), we used a 3′:5′ amplification ratio assessment [28]. This assay aimed at measuring the integrity of an ubiquitin carboxyl-terminal hydrolase blueberry sequence (*UBP14*; Allan Brown, NCSU, personal communication). For this assay, we designed two primer pairs (UBP14_5F, 5′-AGGTGGGCTATATATCGACATGAAC-3′; UBP14_5R, 5′- TCCCAGTCTTCTCAAAATTCCAA-3′; UBP14_3F, 5′-TTCTTTCTCCACTTCATTGACCAA-3′; UBP14_3R, 5′- GTCGCTCTTCAATACCAAACTTGA-3′) to amplify two cDNA fragments, one from the 5′ (101 bp) and one from the 3′ region (101 bp) of the *UBP14* gene. The fragments are 1,769 and 348 bp, respectively, from the 3′ end of the cDNA. The 3′:5′ amplification ratio of the *UBP14* cDNA fragments was calculated using the comparative C_q_ method [29]. All ratios were inside the range of 1.01–3.72 (2.11±0.23; mean ± SEM). Only if ratios were >4.43-fold would RNA quality be deemed inadequate [30]. Therefore, the cDNAs were judged to be suitable for qPCR analysis.

### Primer design, secondary structure control and real-time qPCR assays

Primer sequences were designed to amplify blueberry orthologs to superior reference genes selected from *Arabidopsis* transcriptome microarray data [26]. This included the following genes: TIP41-like protein (*TIP41*), ubiquitin-conjugating enzyme E2 (*PEX4*), SUMO-conjugating enzyme (*UBC9*), basic helix-loop-helix domain-containing protein (*bHLH*), F-box/kelch-repeat protein (*Fbox*), glyceraldehyde 3-phosphate dehydrogenase (*GAPDH*), pentatricopeptide repeat-containing protein (*PPR*), clathrin adapter complexes medium subunit (*CACSa*), ubiquitin-conjugating enzyme (*UBC28*), polyubiquitin (*UBQ3b*), RNA helicase-like (*RH8*), protein phosphatase 2A (*PDF2*) and two sequences encoding hypothetical proteins *Vc4g26410* and *Vc4g16320* (Table S1). All PCR primers were tested for specificity using NCBI's BLAST software [31]. Primers were designed using the following criteria: Tm of 60±1°C and PCR amplicon lengths of 55–120 bp, yielding primer sequences with lengths of 19–27 nucleotides and GC contents of 37–67%. For predicting the secondary structure of the amplicons, we used MFOLD version 3.4 software with default settings of minimal free energy, 50 mM Na^+^, 3 mM Mg^2+^, and an annealing temperature of 60°C [32]. We chose primers that would yield amplicons with minimal secondary structures and melting temperatures that would not hamper annealing (Figure S1). Designed primers were synthesized by Integrated DNA Technologies (Coralville, IA, USA). Table S1 shows the overall mean real-time PCR amplification efficiency of each primer pair (E) estimated from the data obtained from the exponential phase of each individual amplification plot and the equation (1+E) = 10^slope^ using LinReg software and the criteria of including three-five fluorescent data points with R^2^≥0.998 to define a linear regression line [33]. With this method, the E value is derived from the log slope of the fluorescence versus cycle number curve for each particular primer pair. The method does not require standard curves and yields very similar amplification efficiencies compared to methods based on series of template dilutions [34], [35]. PCR reactions were carried out in an IQ5 (Bio-Rad, Hercules, CA, USA) thermal cycler using iQ™ SYBR® Green Supermix (Bio-Rad, Hercules, CA, USA) to monitor dsDNA synthesis. Reactions contained 1 µl of the diluted cDNA as a template and 0.150 µM of each primer in a total volume reaction of 20 µl. Master mix was prepared and dispensed into individual wells using electronic Eppendorf Xplorer® multipipettes (Eppendorf AG, Germany). The following standard thermal profile was used for all PCRs: polymerase activation (95°C for 3 min), amplification and quantification cycles repeated 40 times (95°C for 30 sec, 60°C for 1 min). The specificity of the primer pairs was checked by melting-curve analysis performed by the PCR machine after 40 amplification cycles (60 to 95°C) and is shown in Figure S2. Fluorescence was analyzed using iQ5 2.1 standard optical system analysis software v2.1 (Bio-Rad). All amplification plots were analysed using a base line threshold of 30 relative fluorescence units (RFU) to obtain C_q_ (quantification cycle) values for each gene-cDNA combination.

### Data analysis

To determine which reference genes were best suited for transcript normalization in blueberry, we first used the statistical algorithm geNorm [36]. In a second approach, the coefficient of variation of normalized relative expression levels was calculated based on the formulas (formula 11, 13, 15, 17, 18, 19 and 20) described in the qBase software [17]. In short, C_q_ values were incorporated into an Excel worksheet and transformed into relative quantities (RQs) using the efficiency of each primer pair and the sample with the lowest C_q_ as a calibrator. Then, a sample-specific normalization factor (NF) was estimated as a geometric mean of RQs for the candidate genes. Finally, the mean coefficient of variation (CV) for all reference genes was calculated as the arithmetic mean of the CV estimates of the different reference genes. Data from flower buds and fruits were analyzed separately in both approaches.

### Assessment of normalization

To analyze the reliability of the results based on the set of reference genes here identified, first the influence of the choice of one reference gene on the interpretation of output data was addressed. The expression level of a gene encoding pectate lyase (*PL*), previously described by [22], was quantified at two different stages of development in fruit tissues and was normalized to each of the reference genes using the delta-delta method modified by the efficiency correction as described by [15]. Then, a normalization factor (determined by calculating the geometric mean of the best reference genes) was calculated and their normalized relative quantities were plotted to allow direct comparison between the different samples. Finally, the normalized relative quantities of the *PL* gene over different stages of fruit development were obtained using three normalization factors based on the use of the 2 (NF_2_) or 3 (NF_3_) best-scored references and the use of one single gene (*GAPDH*). Calculations were performed using the advanced quantification model with efficiency correction, multiple reference gene normalization, and use of error propagation rules described by [17].

## Results

### *In silico* highly stable expression from blueberry 454 transcriptome data

**Figure 1:**
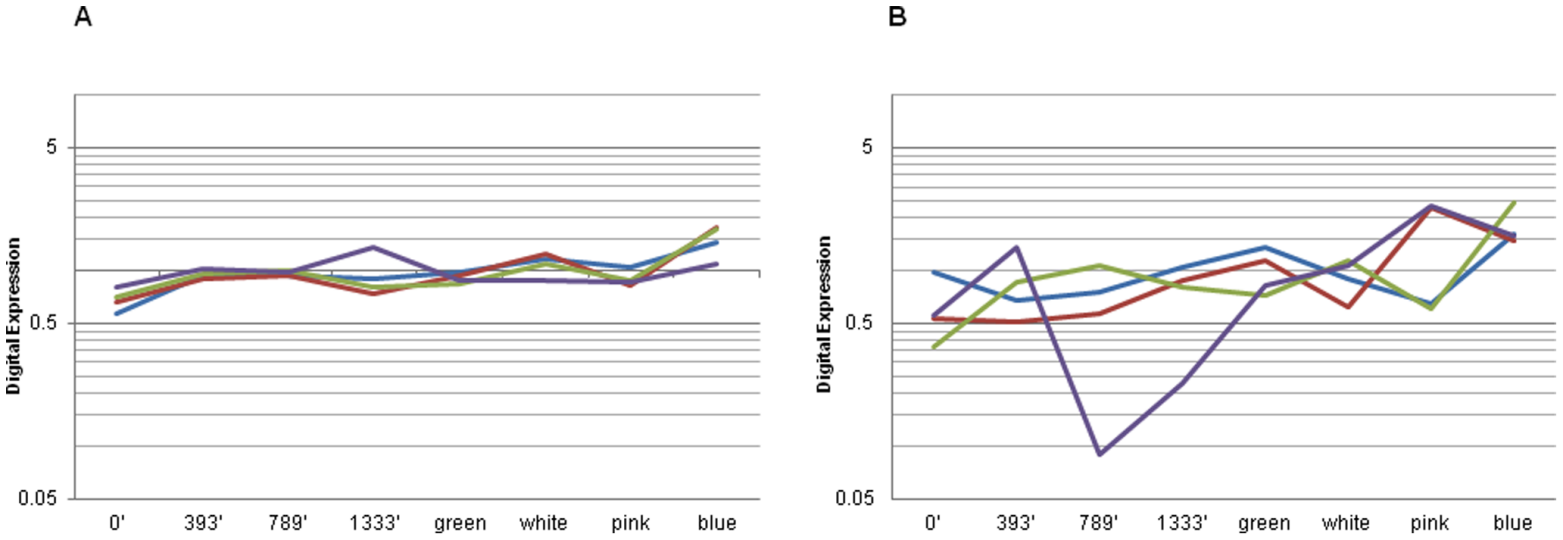
Digital expression level of blueberry genes in dormant floral buds and fruits at different stages of development. **A.** Relative virtual expression of unigenes from the 454 transcriptome database representing putative orthologs to *Arabidopsis* genes *UBC9* (contig02681 and contig14017 with 96% and 93% identity at the amino acid level, respectively; red and blue), *UBC28* (contig13874 with 92% identity; green) and *RH8* (contig01609 with 45% identity; purple). **B.** Relative virtual expression of former frequently used blueberry reference genes in qPCR analyses, i.e. *ACT* (contig00039, red), *metallothionein* (contig0266, purple), *GAPDH* (contig00502, blue) and *EF1a* (contig14280, green). Contig Ids are from the ‘All’ assembly from the transcriptome database [22]. The digital expression for each contig in every sample is expressed relative to the average expression level over all the libraries.

The goal of this research was to find internal reference genes for blueberry expression analyses. We focused on the evaluation and normalization of transcript abundance in flower buds at different stages of cold acclimation and in fruit at different stages of development using qPCR analysis. The *in silico* approach was applied to identify reference genes among different tissues in the following way. First, we mined the large transcriptome data sets for confirmation that highly stable genes were present in the publicly available sequences (http://bioinformatics.towson.edu/BBGD454/). In order to test for likely conservation of gene expression stability across different plant species, we used the set of reference genes described for *Arabidopsis* as a starting point [26]. Putative blueberry orthologs with at least 7 ‘present’ calls across the 8 libraries (flower buds at 0′, 397′, 789′ and 1333′ chill units, and fruit at green, white, pink and blue stages of development) were identified using the Blast algorithm [31]. The number of reads of each contig in each library was determined and their expression profiles were obtained via a digital approach relative to the expression of buds at 0′, which was arbitrarily set as 1. Electronic expression computations (fold expression) were performed as *m*/(*n*[*M*/*N*]), where *m* and *n* are the number of reads from the libraries in the particular contig, and *M* and *N* are the total number of reads in contigs from the libraries that are being compared. Figure 1 shows the relative virtual expression levels of some representative unigenes from this analysis and, for comparison, relative virtual expression of other control genes previously used in blueberry expression analyses. This comparison suggests that some frequently used internal controls in blueberry expression studies may have considerably higher levels of variation than the new potential reference controls and that the transcriptome data contains some interesting candidates for further detailed study.

**Figure 2:**
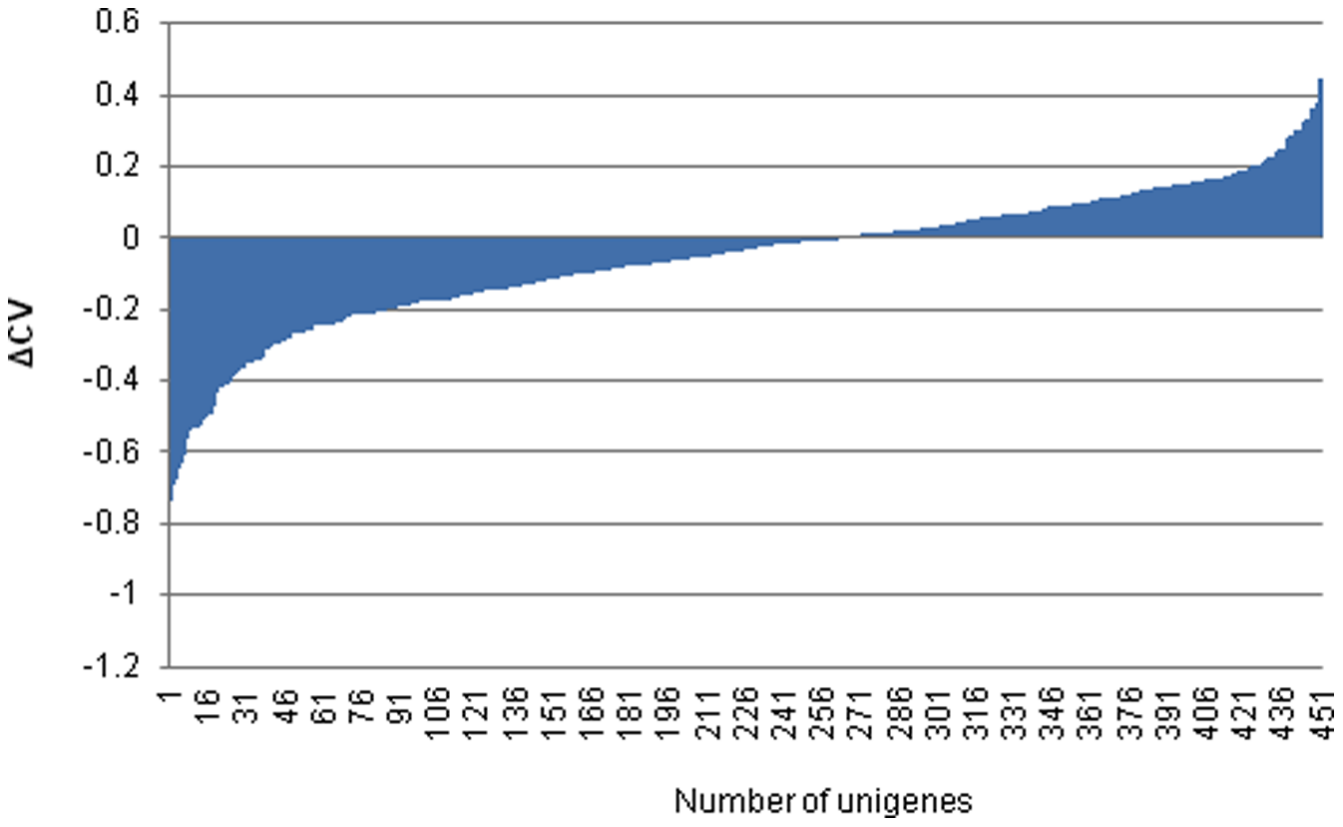
Coefficients of variation predominantly decrease after omission of fruit series. Change of the CV after the omission of the transcriptome fruit libraries over 451 unigenes.

Next, the mean expression value (MV) and the standard deviation (SD) over all experiments were calculated for all unigenes, where at least 7 reads were present in all 8 libraries, followed by the determination for each unigene of the coefficient of variation (CV, calculated by SD/MV). The CV values were analyzed to determine whether the tissue type had a marked influence on the result. Most CVs (262 unigenes out of 451, that is >58%) decreased after omission of the fruit series. Moreover the magnitude of the CV change, after removing the fruit series, was higher for the unigenes that showed a decrease (−0.18±0.010) than for the unigenes that showed an increase in CV (+0.12±0.007). Overall, this suggests that some molecular processes are quite different during flower bud and fruit development and their transcriptomes would be better treated separately in order to identify stable reference genes (Figure 2).

### Experimental selection of reference genes for qPCR

After confirming by digital analysis that identification of potential reference genes with stable expression is possible using the publicly available transcriptome database and that the stability of reference genes might be tissue-dependent, we attempted to keep fine-tuning the selection of the best experimental candidates for qPCR analysis. For this, the blueberry transcriptome was queried to identify putative orthologs of the most stable *Arabidopsis* reference genes, not just those present across all the blueberry libraries. Eighteen genes out of the top 23 *Arabidopsis* genes could be identified in the transcriptome database in at least one library or assembly. After performing the *in silico* quality controls on primer pairs and amplicon sequences described in the Materials and Methods, 10 primer pairs from these genes were retained for the experimental analysis: *TIP41*, *PEX4*, *UBC9*, *bHLH*, *Fbox*, *GAPDH*, *PPR*, *PDF2* and two sequences encoding hypothetical proteins *Vc4g26410* and *Vc4g16320*. The nomenclature adopted for the two sequences with unknown functions was based on the blueberry (*V. corymbosum*) contig that best corresponds to the orthologous *Arabidopsis* gene identifiers. The amino acid identities between the blueberry and *Arabidopsis* sequences were between 36% and 96% and are listed in Table S1. The values found were consistent with those obtained previously in similar comparisons [37], [38], [39]. Another 4 primer pairs amplifying previously reported reference genes in blueberry were also tested, *ubiquitin-conjugating enzyme* (*UBC28*), *RNA helicase-like (RH8*), *clathrin adapter complexes medium subunit family protein* (*CACSa*), and *polyubiquitin* (*UBQ3b*) [40].

### Assessment of gene expression stability using statistical analyses

**Figure 3:**
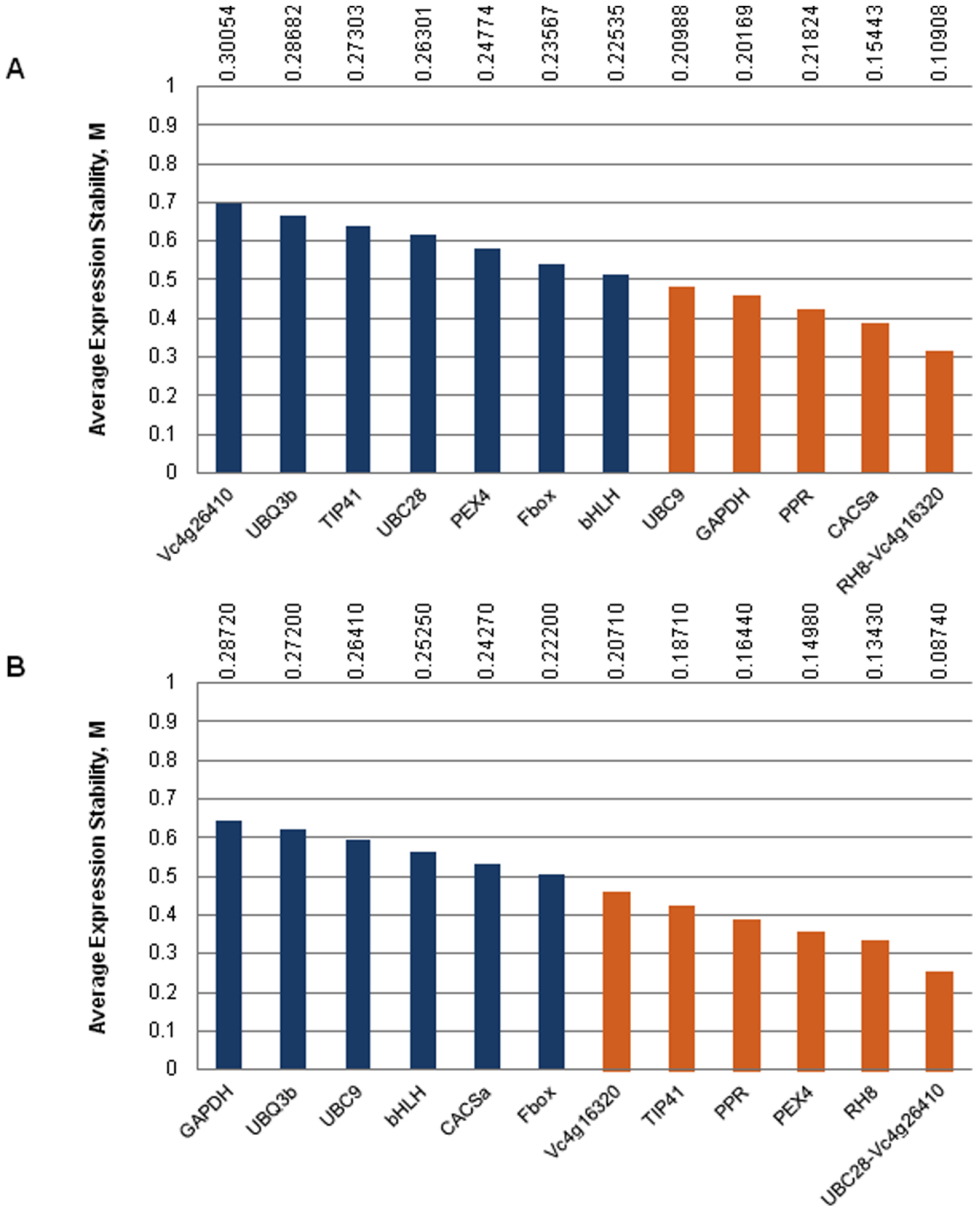
geNorm ranking of 13 reference genes from blueberry series. **A.** Floral bud series **B.** Fruit development series. Vertical numbers at the top indicate the CV values of the reference genes involved in the normalization. References showing highly stable expression (*M* values<0.5) are represented as orange bars.

The C_q_ values were incorporated into an Excel worksheet and transformed into relative quantities (RQs) using the efficiency of each primer pair and the sample with the lowest C_q_ as a calibrator. The *PDF2* assay was discarded from further analysis due to non-specific amplification. The rest of the primers produced a specific PCR product indicated by melting curve analysis (Figure S2). All primer pairs gave amplication efficiencies over 0.88, 10 primer pairs over 0.90 and six primer pairs over 0.95. The efficiencies ranged from 0.8801 (*bHLH*) to 1.0994 (*UBC28*). The relative quantities were treated as input data and the expression levels were analyzed using geNorm software [36]. This determines the *M* value, a measure of the gene expression stability, where *M* is the average pair-wise variation of a particular gene compared with that of all other genes. The lower the *M* value, the more stably expressed is the gene. In a second approach, the mean CVs for all reference genes that could potentially be part of the normalization were calculated according to equations defined in the qBase framework [17]. Figure 3 shows the ranking of the genes tested in our blueberry samples according to their *M* and CV values. In the floral bud series, *RH8* and *Vc4g16320* were the most stably expressed genes (*M* = 0.3172), whereas expression of *Vc4g26410* was quite variable within the panel. On the other hand, in the fruit development series, *UBC28* and *Vc4g26410* were the most stably expressed genes (*M* = 0.2518), while *GAPDH* showed higher expression variability. Without exception, stability values were much lower than the default threshold of 1.5 defined in the algorithm [36]. In addition, *M* values<0.5 were obtained for 6 of the 13 tested reference genes in the floral bud series and 7 genes in the fruit panel. M values in this range indicate highly stable expression. Genes with values inside the optimal range for homogeneous sample panels (*M* and CV values lower than 0.5 and 25% respectively according to [17], were the most stably expressed and included: *RH8*, *Vc4g16320*, *CACSa*, *PPR*, *GAPDH* and *UBC9* (*M* = 0.483 and CV = 0.210) for floral buds; *UBC28*, *Vc4g26410, RH8, PEX4, PPR, TIP41and Vc4g16320* (*M* = 0.460 and CV = 0.207) for fruits.

### Single reference-based normalization

**Figure 4:**
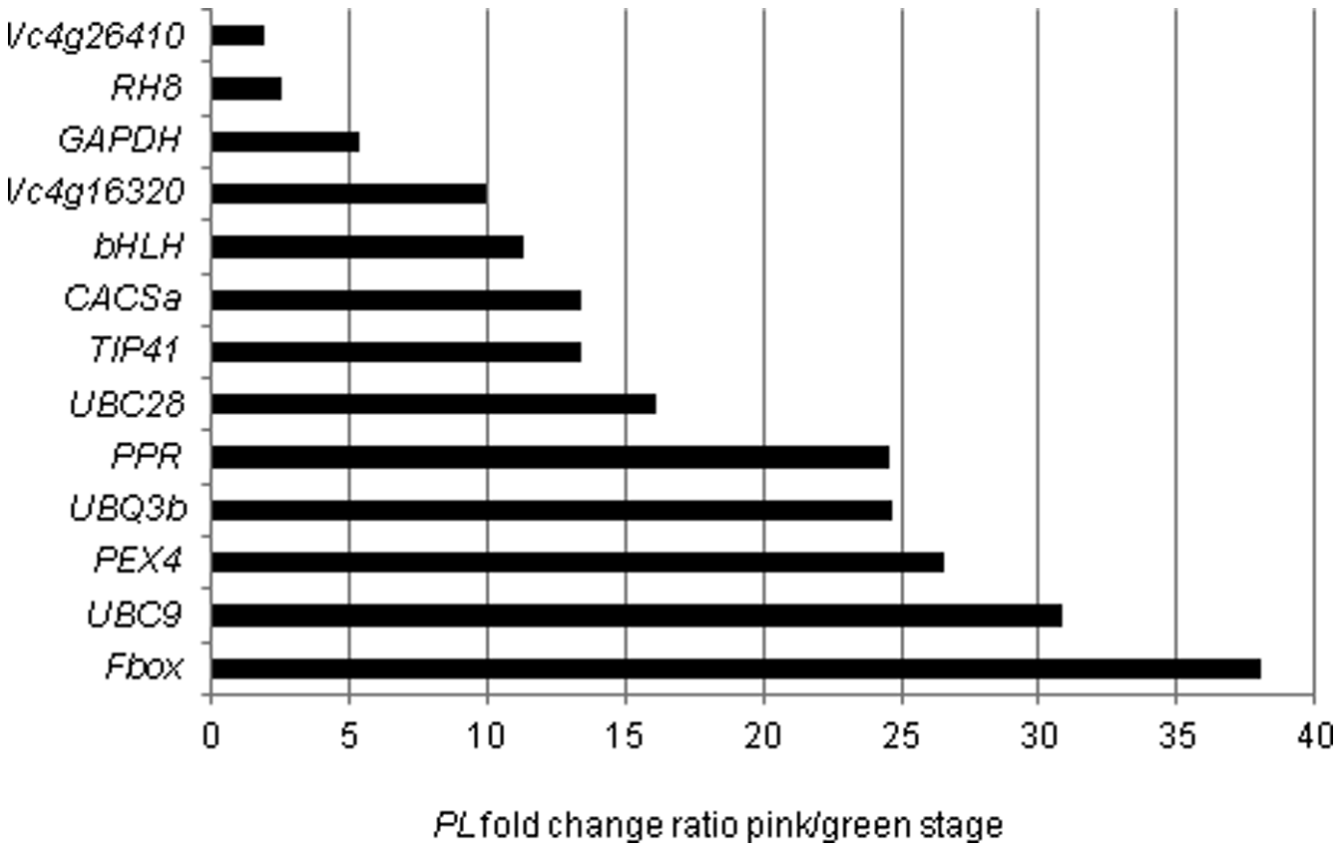
Normalization of pectate lyase (*PL*) mRNA expression in blueberry at green and pink fruit developmental stages using 13 different reference genes. The *PL* mRNA ratio in pink fruit: green fruit (x-axis) was calculated using the 13 different reference genes shown on the y-axis. The ratio was calculated taking the gene specific amplification efficiencies into account (target gene and reference gene) according to the quantification model proposed by Pfaffl [15].

Since many scientists still use a single reference gene in qPCR analyses, next we quantified the ratio of the *pectate lyase* gene (*PL*) at green and pink fruit developmental stages relative to each of the 13 reference genes (Figure 4), in order to determine how normalization using only one reference gene can compromise the results. Normalization of *PL* mRNA with the various references did not give contradictory results, as all ratios indicated up-regulation at the pink fruit stage (ratio>1-fold). However, the ratios obtained were very different depending on the reference gene used. The average *PL* mRNA ratio (pink fruit: green fruit) was 16.84-fold, but it ranged from 1.93-fold to 38.07-fold depending only on the normalizer chosen.

### Validation of putative references genes

**Figure 5:**
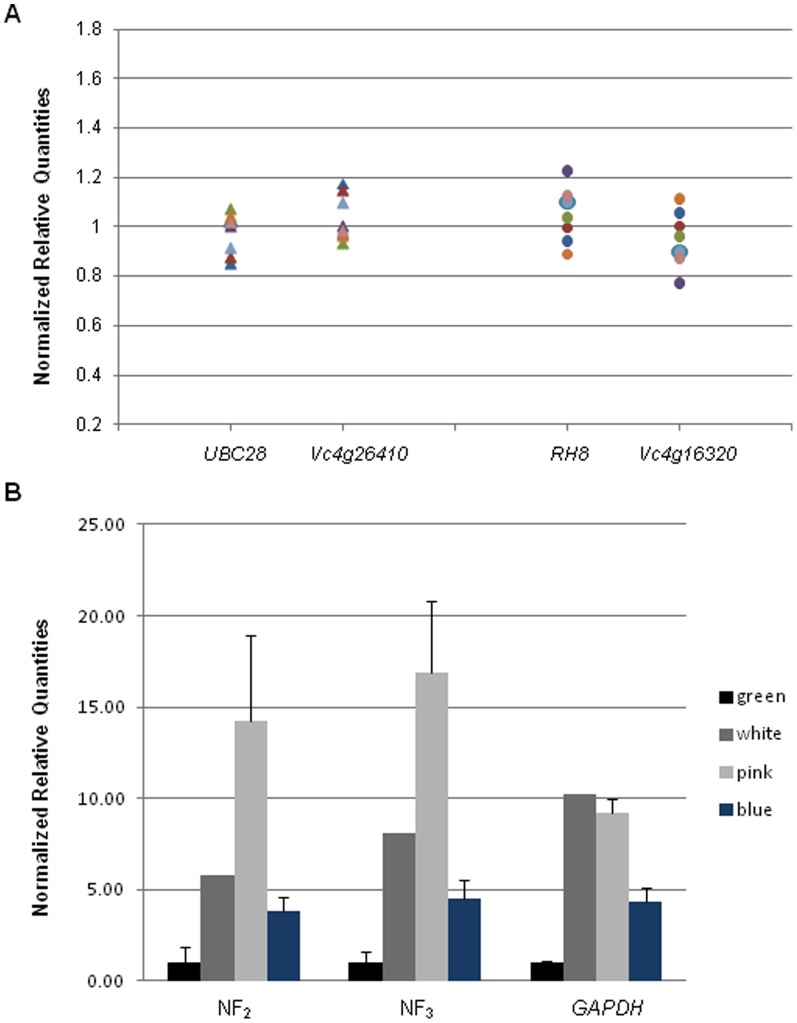
Evaluation of reference genes for blueberry. **A.** To show the stability of the reference genes, the normalized relative quantities for the best references were calculated in each sample. Fruit samples are represented by triangles and floral buds by circles. **B.** Normalized relative quantities of *PL* gene in fruits over four development stages. Three normalization approaches were used: the use of the two or three best-scored genes (NF) and use of a single gene (*GAPDH*) as normalizer. NRQ were rescaled to green that was arbitrarily set as 1.

To test the putative reference genes identified above, we plotted the normalized relative quantities of the two best reference set compared to the overall geometric mean of the three best scored genes according to geNorm. The average fold expression difference was only 1.004±0.0023 and 1.000±0.0296 for the fruit samples and flower buds, respectively. Maximum difference between samples was detected in floral buds with 0.34-fold change (Figure 5A). In a second attempt to validate the stability of the references, we monitored the mean expression of one target gene during fruit development in the cultivar Bluecrop. Normalized relative quantities of the *PL* gene were obtained using three normalization factors–the use of the 2 (NF_2_) or 3 (NF_3_) best-scored references and the use of one single gene (*GAPDH*). Figure 5B shows transcript levels across four development stages (green, white, pink and blue or ripe fruit). Results showed the same trend when we normalized data using, either the NF_2_ or NF_3_ approach, low transcript levels at the green fruit stage, highest transcript levels at the pink fruit stage, and lower levels at the blue fruit stage. However, when we used only *GAPDH* as the reference gene, the expression of the *PL* gene was high at both the white and pink fruit stages and actually peaked at the white fruit stage rather than the pink fruit stage. Finally, we explored the stability values found by testing the references recommended for fruits and floral buds in a third tissue: leaves from Bluecrop and Tifblue genotypes collecting at 5 time points during the summer 2012. Data were analyzed through the two statistical approaches described above and high expression stability was obtained here as well (Figure S3).

## Discussion

The advent and adoption of genomic technologies in the field of plant research will allow researchers to address fundamental questions of genome biology and issues related to complex agronomic traits in ways that have not been possible before [41]. Our laboratory has been working toward increasing our understanding of complex molecular regulation networks in blueberry such as the genetic control of cold hardiness, chilling requirement, and fruit quality traits [21]. Recently, new collections of blueberry transcriptome sequences based on high-throughput technologies have been released [22], [42], [43]. They represent a substantial improvement to the limited genomic resources that existed before in the species and will provide novel insights into various biological processes [44]. Quantitative real-time PCR is the technique of choice to validate gene expression results derived from the above-mentioned high-throughput methods. It is necessary, however, to understand the principles underlying the technology and how the entire workflow influences the reliability of the conclusions [3], [45]. These problems have been known and publicized for quite a long time, and the plant scientific community has gradually recognized the need for more robust standards [11], [12], [13], [14], [37], [46]. One subject of considerable controversy has been the method of data normalization. There is now a compelling body of literature that accurate normalization is fundamental for reliable qPCR results and an availability of stable validated reference genes is a necessary prerequisite to any quantitative analysis.

In this study, we analyzed the expression of 13 reference genes across 32 cDNA samples derived from two different tissues, fruit and flower buds. This represents considerable initial variability because of the broad physiological and cellular changes that occur during fruit development and during chill unit accumulation of floral buds over the winter. Moreover, we used two different genotypes that indeed represent two different blueberry species (*V. corymbosum* or highbush blueberry and *V. virgatum* or rabbiteye blueberry). To screen novel superior cross-species reference genes in blueberry, we first mined the transcriptome database described by [22], to identify putative orthologs of known *Arabidopsis* reference genes that have been shown to be the most stable in a developmental series [26]. From an *in silico* analysis, we found a set of unigenes with potentially more stable expression than those frequently used for qPCR normalization. Because we limited this first analysis to only those sequences present in all libraries, we decided to then extend the identification of orthologs to the whole blueberry transcriptome. The idea of selecting putative orthologs of known reference genes from other species was an original hypothesis proposed by [26]. This study was of notable importance because it led to the identification of superior reference genes compared with those that were available previously. The approach of identifying better reference genes from the orthologs of other plant species that were known to be stably expressed has proved to be more efficient than that of randomly testing endogenous genes [11], [37], [47], [48].

Factors known to affect the reliability of gene expression data such as RNA quality, DNase treatment, two-step RT-qPCR, the use of the same RT master mix that generated one cDNA batch, primer design keeping in mind the presence of secondary structures in the amplicon, efficiency correction, and non-specific amplification were controlled during the experiment. The geNorm software tool [36] represents one of the most commonly used algorithms to evaluate candidate reference genes by their average gene transcript expression stability across samples and has established itself as the *de facto* standard method [3], [49]. In a second approach, the CV of normalized relative quantities was determined based on the equations defined by the qBase framework [17]. Thus, we identified a significant number of reference genes with high stability values for both fruits and floral bud tissues. In general, the results showed a considerable number of genes with acceptable values in terms of stability, with the worst ranked genes having *M* values of 0.696 and 0.644 for buds and fruit organs, respectively. Although there were some similarities, in general the genes showed different regulation depending on the tissue analyzed. This result is concordant with the *in silico* analysis that suggested that the flower bud and fruit series transcriptomes are very different; therefore, probably different reference genes would be optimum for each tissue type (Figure 2). From the top 5 references, *RH8* and *PPR* were shared by both tissues. If we define acceptable reference genes as those with stability values of *M*<0.5 and CV<0.25 [17], a third reference gene *Vc4g16320* was found under both experimental panels. Conversely, *GAPDH* constituted one of the top 5 reference genes for flower buds and *Vc4g26410* for the fruit development stages but they showed differential expression for the other experimental panel, having the lowest stability value of those tested. The two references tested that had high stability values *RH8* (buds and fruits) and *UBC28* (fruits) were not part of the initial set provided by [26], instead they were recently identified as stably expressed in an experimental panel made of rabbiteye and southern highbush blueberry samples across multiple organs [40]. Therefore, our data confirms their use as stable reference genes and these results combined with those of Vashisth et al. [40] provide a comprehensive set of stable references under different conditions in blueberry. In addition, we confirmed the stable expression of these genes in leaf tissue from the two cultivars.

One of the most intriguing issues in the field of qPCR relates to normalization using only one single unvalidated reference gene. Hugget and Bustin [45] noted that a squalid 10.5% of qPCR analyses recently published in three leading high-impact journals used more than one single reference gene. Despite the vast number of publications showing that the use of a single reference gene yields unreliable data, this approach is still routinely used in plant research. Even working with relatively stable genes across a given sample, as in our case study here, we found that differences in expression levels of ∼20-fold can be generated based on only the choice of reference gene. This analysis clearly demonstrates that instead of accomplishing the goal of normalization, that is, removing technical variation, the use of a single reference gene can actually add more variation and lead to confounding conclusions. This also highlights the highly improbable chance that small differences in gene expression can be detected based on the sole use of one reference gene.

Finally, to test the suitability of the genes recommended in the present study, we used the same set of references for normalization by calculating the geometric mean of the three best control genes in each panel and plotting their normalized relative quantities across the samples showing that only a small maximum difference between samples could be detected (0.34-fold change in floral buds) and that the values were closely distributed around 1-fold. This is a straightforward, rapid method that can be useful in evaluating potential reference genes before they are actually used in experimentation. Moreover, we analyzed the expression pattern of the *PL* gene that has been shown to play an important role in fruit softening by degradation of the primary cell wall and middle lamella during ripening [50]. When the two or three best reference genes were utilized for calculating the normalization factor, the expression level of *PL* mRNA increased gradually until it peaked at the pink fruit stage and then decreased about 3-fold during the ripe stage. When we used the worst single reference gene in our panel for normalization, the profile was similar except that *PL* mRNA expression peaked at the white stage rather than the pink stage. Expression peaking at the white fruit stage is likely to be an artifact caused by the intrinsic variation of the gene *GAPDH* used for normalization (Figure 5B), since expression patterns obtained with the two or three best reference genes both showed up-regulation of *PL* at the pink stage.

In summary, this study provides ample possible reference genes for use in blueberry experimental research based on transcriptome data mining and experimental validation. The likelihood that one orthologous reference gene will work well in a given species seems to decrease as the distance between the species increase phylogenetically. Nevertheless, there is cumulative evidence that gene expression stability is higher for a given developmental process between distinct species than for distinct developmental processes within a given single species [37], [47], [48]. Depending on the tissues analyzed, the recommended blueberry reference genes, *Vc4g16320*, *CACSa*, *PPR*, *GAPDH*, *UBC9*, *Vc4g26410*, *PEX4* and *TIP41*, were also among the highest stably expressed genes in *Arabidopsis*. This suggests these genes may be useful for related members of the *Ericaceae* as well, such as cranberry and rhododendron, where molecular knowledge is more limited and the availability of reference genes is a major point of concern.

## Supporting Information

Figure S1 **Modeling of secondary structures of the amplicons for the assays designed in this study.** Thermodynamic stability (ΔG in kcal/mole) is presented in the figures. Primers are indicated by blue arrows. Although some secondary structures might be present where primers anneal for some assays, they have a positive ΔG value and Tm<60°C and hence will not influence the amplification efficiency.
(TIFF)

Figure S2 **Dissociation curves for 12 representative PCR products.**
(TIFF)

Figure S3 **geNorm ranking of 10 reference genes from blueberry leaves.** Vertical numbers at the top indicate the CV values of the reference genes involved in the normalization. References showing highly stable expression (*M* values<0.5) are represented as orange bars.
(TIFF)

Table S1 Description of reference genes, Arabidopsis Gene Index (AGI) orthologous identifiers and blueberry primer sequences. Primer PCR efficiency and PCR Tm product data represent mean values ± SE. PCR efficiencies (E) calculated according to the equation (1+E) = 10^slope^
(PPTX)
