# Supplementary figures and images for: Superior Cross-Species Reference Genes: A Blueberry Case Study
Source: PLoS One. 2013 Sep 18;8(9):e73354. doi: 10.1371/journal.pone.0073354 (PMC3776805; doi:10.1371/journal.pone.0073354)

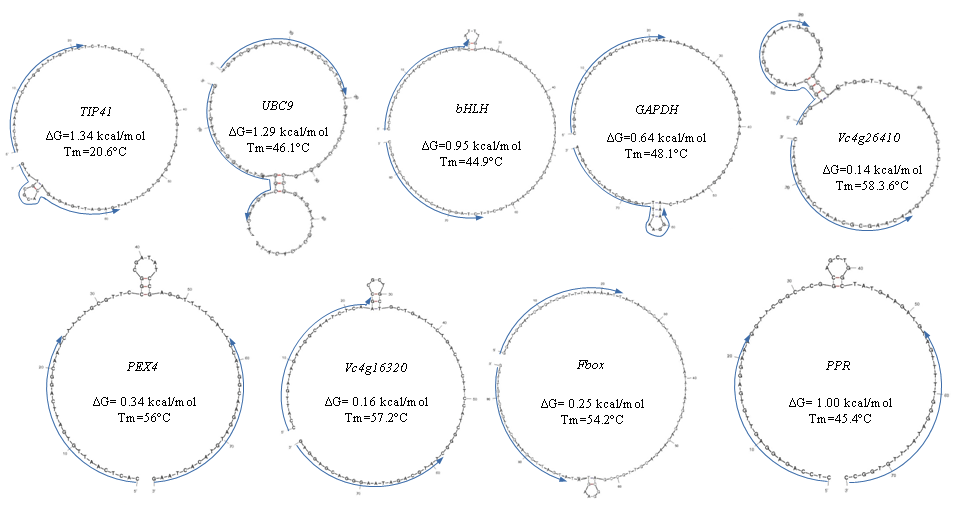

Supplement: Figure S1 — Modeling of secondary structures of the amplicons for the assays designed in this study. Thermodynamic stability (ΔG in kcal/mole) is presented in the figures. Primers are indicated by blue arrows. Although some secondary structures might be present where primers anneal for some assays, they have a positive ΔG value and Tm<60°C and hence will not influence the amplification efficiency. (TIFF) [file pone.0073354.s001.tiff]

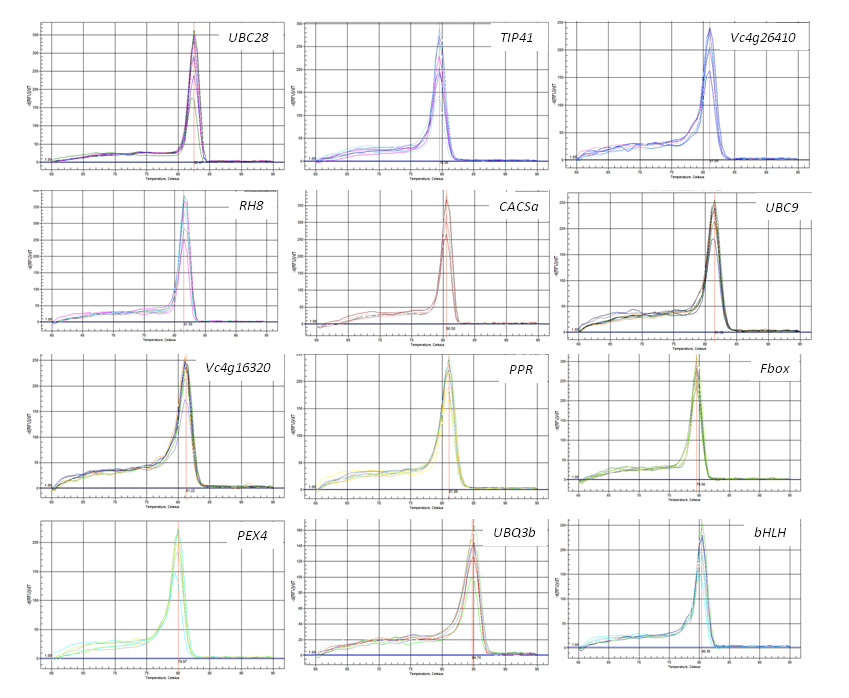

Supplement: Figure S2 — Dissociation curves for 12 representative PCR products. (TIFF) [file pone.0073354.s002.tiff]

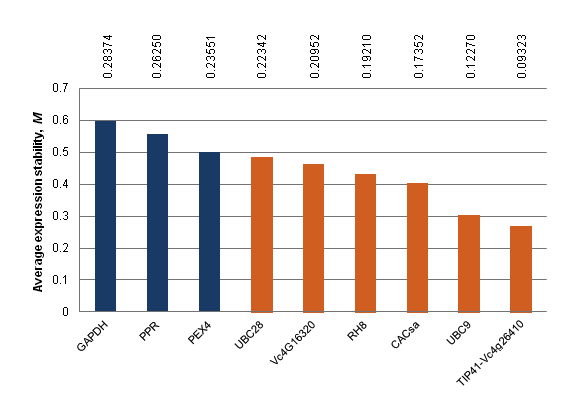

Supplement: Figure S3 — geNorm ranking of 10 reference genes from blueberry leaves. Vertical numbers at the top indicate the CV values of the reference genes involved in the normalization. References showing highly stable expression (M values<0.5) are represented as orange bars. (TIFF) [file pone.0073354.s003.tiff]
