# Supplementary material for: Superior Cross-Species Reference Genes: A Blueberry Case Study
Source: PLoS One. 2013 Sep 18;8(9):e73354. doi: 10.1371/journal.pone.0073354 (PMC3776805; doi:10.1371/journal.pone.0073354)
Supplement: Table S1 — Description of reference genes, Arabidopsis Gene Index (AGI) orthologous identifiers and blueberry primer sequences. Primer PCR efficiency and PCR Tm product data represent mean values ± SE. PCR efficiencies (E) calculated according to the equation (1+E) = 10slope (PPTX) [file pone.0073354.s004.pptx]

## Slide 1
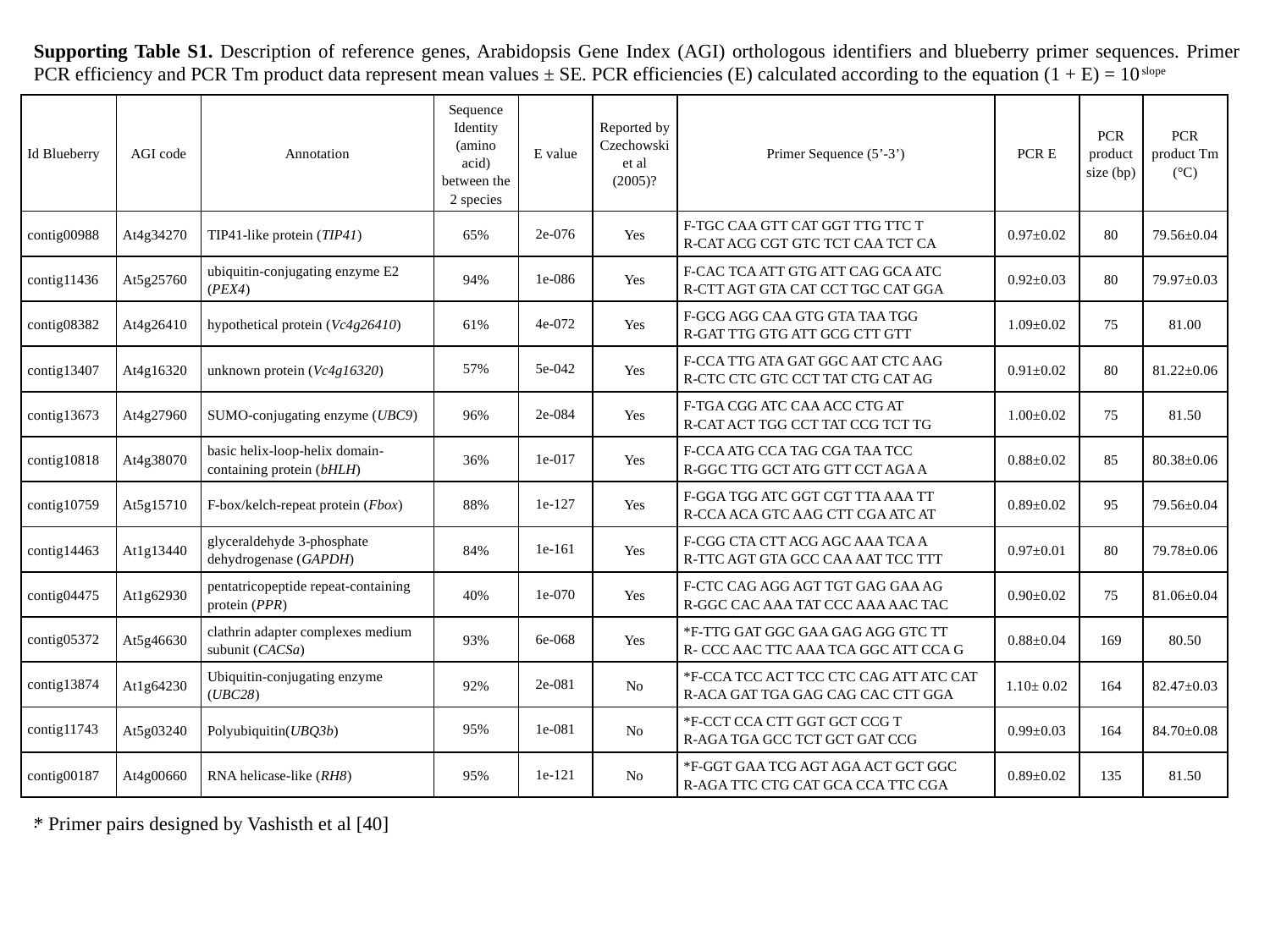

Supporting Table S1. Description of reference genes, Arabidopsis Gene Index (AGI) orthologous identifiers and blueberry primer sequences. Primer PCR efficiency and PCR Tm product data represent mean values ± SE. PCR efficiencies (E) calculated according to the equation (1 + E) = 10slope
| Id Blueberry | AGI code | Annotation | Sequence Identity (amino acid) between the 2 species | E value | Reported by Czechowski et al (2005)? | Primer Sequence (5’-3’) | PCR E | PCR product size (bp) | PCR product Tm (°C) |
| --- | --- | --- | --- | --- | --- | --- | --- | --- | --- |
| contig00988 | At4g34270 | TIP41-like protein (TIP41) | 65% | 2e-076 | Yes | F-TGC CAA GTT CAT GGT TTG TTC T R-CAT ACG CGT GTC TCT CAA TCT CA | 0.97±0.02 | 80 | 79.56±0.04 |
| contig11436 | At5g25760 | ubiquitin-conjugating enzyme E2 (PEX4) | 94% | 1e-086 | Yes | F-CAC TCA ATT GTG ATT CAG GCA ATC R-CTT AGT GTA CAT CCT TGC CAT GGA | 0.92±0.03 | 80 | 79.97±0.03 |
| contig08382 | At4g26410 | hypothetical protein (Vc4g26410) | 61% | 4e-072 | Yes | F-GCG AGG CAA GTG GTA TAA TGG R-GAT TTG GTG ATT GCG CTT GTT | 1.09±0.02 | 75 | 81.00 |
| contig13407 | At4g16320 | unknown protein (Vc4g16320) | 57% | 5e-042 | Yes | F-CCA TTG ATA GAT GGC AAT CTC AAG R-CTC CTC GTC CCT TAT CTG CAT AG | 0.91±0.02 | 80 | 81.22±0.06 |
| contig13673 | At4g27960 | SUMO-conjugating enzyme (UBC9) | 96% | 2e-084 | Yes | F-TGA CGG ATC CAA ACC CTG AT R-CAT ACT TGG CCT TAT CCG TCT TG | 1.00±0.02 | 75 | 81.50 |
| contig10818 | At4g38070 | basic helix-loop-helix domain-containing protein (bHLH) | 36% | 1e-017 | Yes | F-CCA ATG CCA TAG CGA TAA TCC R-GGC TTG GCT ATG GTT CCT AGA A | 0.88±0.02 | 85 | 80.38±0.06 |
| contig10759 | At5g15710 | F-box/kelch-repeat protein (Fbox) | 88% | 1e-127 | Yes | F-GGA TGG ATC GGT CGT TTA AAA TT R-CCA ACA GTC AAG CTT CGA ATC AT | 0.89±0.02 | 95 | 79.56±0.04 |
| contig14463 | At1g13440 | glyceraldehyde 3-phosphate dehydrogenase (GAPDH) | 84% | 1e-161 | Yes | F-CGG CTA CTT ACG AGC AAA TCA A R-TTC AGT GTA GCC CAA AAT TCC TTT | 0.97±0.01 | 80 | 79.78±0.06 |
| contig04475 | At1g62930 | pentatricopeptide repeat-containing protein (PPR) | 40% | 1e-070 | Yes | F-CTC CAG AGG AGT TGT GAG GAA AG R-GGC CAC AAA TAT CCC AAA AAC TAC | 0.90±0.02 | 75 | 81.06±0.04 |
| contig05372 | At5g46630 | clathrin adapter complexes medium subunit (CACSa) | 93% | 6e-068 | Yes | \*F-TTG GAT GGC GAA GAG AGG GTC TT R- CCC AAC TTC AAA TCA GGC ATT CCA G | 0.88±0.04 | 169 | 80.50 |
| contig13874 | At1g64230 | Ubiquitin-conjugating enzyme (UBC28) | 92% | 2e-081 | No | \*F-CCA TCC ACT TCC CTC CAG ATT ATC CAT R-ACA GAT TGA GAG CAG CAC CTT GGA | 1.10± 0.02 | 164 | 82.47±0.03 |
| contig11743 | At5g03240 | Polyubiquitin(UBQ3b) | 95% | 1e-081 | No | \*F-CCT CCA CTT GGT GCT CCG T R-AGA TGA GCC TCT GCT GAT CCG | 0.99±0.03 | 164 | 84.70±0.08 |
| contig00187 | At4g00660 | RNA helicase-like (RH8) | 95% | 1e-121 | No | \*F-GGT GAA TCG AGT AGA ACT GCT GGC R-AGA TTC CTG CAT GCA CCA TTC CGA | 0.89±0.02 | 135 | 81.50 |
.
* Primer pairs designed by Vashisth et al [40]
